# Supplementary material for: Chromothripsis during telomere crisis is independent of NHEJ, and consistent with a replicative origin
Source: Genome Res. 2019 May;29(5):737–49. doi: 10.1101/gr.240705.118 (PMC6499312; doi:10.1101/gr.240705.118)
Supplement: Supplemental Material [file supp_gr.240705.118_Supplemental_file_1.zip › contigs/annotated_contigs/DB88/contig.2.DB88_length_411_mean_cov_5.10462287105.docx]

**DB88_length_411_mean_cov_5.10462287105**

```
AAAAATCCTTTGAGACTACTTATAACAAAAGACATAAAGTCAAAATCACATGGCCTTTTAAATATACCATGTGCAGGCCAGATGTGATG
 >chr1:35856410-35856659 + E=2e-138
GTTCATGCCTGTAATCCCACCGCCTTTGGAATGCTGAGGTGGGAGGACGGCTTGAGGCCAGGAGTTCAGGGGTGGAGTGAGCGGTGACC

ACAGCACAGCAATCCCAGCTGGATGATATAGTGAGACCCTGCCTCTAAAAAATTAAAATAAAATAAAAC|TA|AGGTGTTCAACAAAAA
                                                                      >chr1:35855699-35
AAGCTAAATGTTATTGATACAGAATACAAGGAAACTGAGGTTTCACGAAATTAATGACAACTGTTTACACAGTTCCTGATCTGTAAAAG
855863 - E=3e-84
AACAGTAGTTCCTAGTATTGACCAAAGACTCCATTCAGTACCTAAGGTTAATTTTAC
```
